# Agreement between Patient-reported Pain Medication Use and Electronic Medical Record Data in Surgical Amputation Patients

**DOI:** 10.1097/GOX.0000000000005415

**Published:** 2023-11-27

**Authors:** Carrie A. Kubiak, Jennifer C. Lee, Jennifer B. Hamill, H. Myra Kim, Randy S. Roth, Paul S. Cederna, Michael E. Geisser, Theodore A. Kung, Stephen W. P. Kemp

**Affiliations:** From the * Department of Surgery, Section of Plastic Surgery, Michigan Medicine, Ann Arbor, Mich.; † Center for Statistical Consulting & Research, The University of Michigan, Ann Arbor, Mich.; ‡ Department of Physical Medicine and Rehabilitation, The University of Michigan, Ann Arbor, Mich.; § Department of Biomedical Engineering, The University of Michigan, Ann Arbor, Mich.

## Abstract

**Background:**

Opioid misuse after surgery remains a public health crisis in the United States. Recent efforts have focused on tracking pain medication use in surgical populations. However, accurate interpretations of medication use remain quite challenging given inconsistent usage of different datasets. The purpose of this study was to investigate the agreement between electronic medical records (EMR) versus patient self-reported use of pain medications in a surgical amputation population.

**Methods:**

Patients undergoing major lower extremity amputation or amputation-related procedures were included in this study. Both self-reported and EMR data for pain medication intake were obtained for each patient at three time points (preoperatively, 4 months postoperatively, and 12 months postoperatively). Percentage agreement and the kappa statistic were calculated for both usage (yes/no) and dose categories.

**Results:**

Forty-five patients were included in this study, resulting in 108 pairs of self-reported and EMR datasets. Substantial levels of agreement (>70% agreement, kappa >0.61) for opioid use was seen at preoperative and 12 months postoperative. However, agreement dropped at 4 months postoperatively. Anticonvulsant medication showed high levels, whereas acetaminophen showed lower levels of agreements at all time points.

**Conclusions:**

Either self-reported or EMR data may be used in research and clinical settings for preoperative or 12-month postoperative patients with little concern for discrepancies. However, at time points immediately following the expected end of acute surgical pain, self-reported data may be needed for more accurate medication reporting. With these findings in mind, usage of datasets should be driven by study objectives and the dataset’s strength (eg, accuracy, ease, lack of bias).

**Takeaways**

**Question:** We aimed to investigate if an agreement exists between electronic medical records versus patient self-reported use of pain medications in a surgical amputation population.

**Findings:** In total, 45 patients were included, resulting in 108 pairs of data analyzed. Substantial levels of agreement (>70% agreement, kappa >0.61) for opioid use was seen at preoperative and 12 months postoperative.

**Meaning:** Self-reported or electronic medical records data may be used in research and clinical settings for preoperative or 12-month postoperative patients, with self-reported data potentially more accurate.

## INTRODUCTION

The opioid epidemic remains a pervasive issue in the United States with almost 50,000 deaths attributed to opioid overdoses in 2019 alone.^1^ Despite these numbers, opioids remain one of the most prescribed medications for postoperative pain management, with 91.6% of surgical patients in the United States being prescribed an opioid at the time of discharge.^2,3^ These surgical patients have been found to be at risk for prolonged or chronic opioid use after the acute postoperative period.^4,5^ For these reasons, there is increasing focus on multimodal analgesia regimens as a method of mitigating opioid prescribing and patient opioid intake.^6,7^

Coincident with improving stewardship in pain medication prescribing is increasing emphasis on the accurate assessment of postoperative pain medication use among patients.^8,9^ However, methods of quantifying pain medication use in these patient populations have been inconsistent. Some studies use surveys or self-reported patient data,^10,11^ whereas others use medication lists found in electronic medical records (EMR).^12,13^ Both methods have their own benefits and limitations. Self-reported data have the main advantage of potentially providing more accurate data regarding actual medication consumption, even if different than prescribed.^14^ Disadvantages of self-reported data are mainly associated with retrieving the information, recall bias,^14^ or bias of patients producing only socially acceptable responses.^15^ Conversely, the main advantage of medical record data is the abundance of historical data in chronological order and ease of access.^14^ The biggest disadvantage of medical records is that the data may be incomplete when over-the-counter (OTC) drugs are not documented, or in the case of multiple prescribing providers with separate medical records containing different pieces of information.^14^

There is a paucity of research examining the agreement between self-reported and EMR medication intake data in the surgical population. With the inconsistencies in pain medication use quantification, it is difficult to draw conclusions across different studies without this agreement data. The purpose of this study was to assess the agreement between patient self-reported and EMR pain medication use in a surgical amputation population. This study examined the agreement in medication use (yes/no), dose category, and numeric dosage for three commonly used medication types for treating pain in a surgical amputation population: opioids, acetaminophen, and anticonvulsants.

## METHODS

IRB approval for this study was obtained through the University of Michigan institutional review board (HUM00120915). Major lower extremity amputation adult patients enrolled in a prospective study on postamputation pain were eligible for this study and had a history of either unilateral below-knee, through-knee, or above-knee amputation from any cause.

**Fig. 1.**
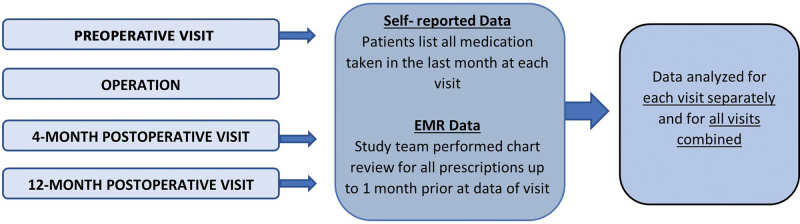
Schematic of data collection for medication intake.

Self-reported medication data were obtained with patient surveys. Subjects were asked to list all prescribed and OTC medications that they had taken over the previous month. Instructions encouraged participants to record *exactly* how they were taking the medication, even if it differed from how it was originally prescribed. Self-reported data were collected at three time points: preoperatively, 4 months postoperatively, and 12 months postoperatively (Fig. 1). Given the original aim of the prospective study was to assess chronic pain, and chronic pain is defined as pain lasting longer than 3 months, the 4-month and 12-month postoperative time points were specifically chosen.^16^ Medication name, dose, and frequency were recorded for each medication.

The same EMR data were obtained via patient chart review within a 1-month window around the time points of patient-reported data. The institutional EMR data include all controlled substances, OTC, and other multimodal pain prescriptions, and are separate from the Michigan Automated Prescription System. For both self-reported and EMR data, if dosage was presented as a range or as a “PRN” (*pro re nata* or “as needed”), the highest possible dosage was recorded. For example, “oxycodone 5mg 1-2 tabs Q4-6H PRN” would be considered “oxycodone 10mg Q4H” or 60mg oxycodone per day. Prescriptions issued and active up to 1 month before each date of the self-reported surveys were included in EMR medication data, but medications prescribed after the survey date were excluded.

Self-reported and EMR medication data were then organized using the Neuropathic Pain Medication Log (NPML). The NPML was developed for this study as a comprehensive method for identifying and quantifying pain medication for patients with major limb amputations. It classifies pain medications into several types, including OTC analgesics (eg, acetaminophen), opioids (including tramadol), and anticonvulsants (including carbamazepine, gabapentin, oxcarbazepine, and pregabalin). After the categorization, the NPML reports the total number of specific medications, daily medication dosage, and dose category for each medication type from both patient self-reported and EMR data.

### Data Analysis

Self-reported and EMR medication data were analyzed for each separate time point and for all three time points combined. Analysis of the separate time points was done to assess if agreement differs based on different time points preoperatively and postoperatively, whereas the combined analysis was performed to assess overall agreement for comparison to medication dataset agreements in the literature. Individual medications were recorded as taking (yes) or not taking (no). Data for acetaminophen, opioid, and anticonvulsant medication types were further categorized into daily dose categories (low dose, medium dose, high dose). Opioid dose categories were defined as per Edlund and colleagues^17^: low daily dose (1–36 mg per day), medium daily dose (36–120 mg per day), and high daily dose (>120 mg per day). Dose categories for other medications were determined with the assistance and expertise of a pain clinical pharmacist specialist and are displayed in Table 1.

**Table 1.** Agreement between Patient Self-reported and EMR Medication Usage by Medication Type

Preoperative					4 Months Postoperative					12 Months Postoperative				
	**Self-reported**					**Self-reported**					**Self-reported**			
**EMR**	**No**	**Yes**	**%A**	**Κ**	**EMR**	**No**	**Yes**	**%A**	**Κ**	**EMR**	**No**	**Yes**	**%A**	**Κ**
**Opioid**					**Opioid**					**Opioid**				
No	22	3	84%	0.68	No	16	1	68%	0.40	No	13	0	92%	0.84
Yes	4	16			Yes	11	10			Yes	2	10		
**Acetaminophen**					**Acetaminophen**					**Acetaminophen**				
No	19	6	73%	0.46	No	12	3	63%	0.29	No	11	1	64%	0.29
Yes	6	14			Yes	11	12			Yes	8	5		
**Anticonvulsants**					**Anticonvulsants**					**Anticonvulsants**				
No	27	1	96%	0.91	No	10	1	89%	0.76	No	6	1	88%	0.71
Yes	1	16			Yes	3	24			Yes	2	16		

Percentage agreements and kappa statistics were then calculated for medication use and dose category across all medication types. Kappa statistics were interpreted as either slight agreement (0–0.20), fair (0.21–0.40), moderate (0.41–0.60), substantial (0.61–0.80), or near-perfect (>0.80) agreement, with a minimally acceptable kappa of 0.61.^18^ Numeric dosage data were then presented in scatterplots to show the relationship between self-reported and EMR numeric dosage data for each specific medication. Spearman correlation coefficient was used to examine the correlation between self-reported dose and EMR. Interpretation of Spearman correlation coefficients was as follows: ±1, perfect association; ±0.8–0.9, very strong association; ±0.6–0.7, moderate association; ±0.3–0.5, fair association; ±0–0.2, no or poor association.^19^ All analyses were done using R (R Core Teams, version 4.2.0); package vcd (Meyers 2021, R package version 1.4-9) was used for kappa analysis and package ggplot2 for figure production.

## RESULTS

A total of 108 pairs of self-reported and EMR data were included in this study. Forty-five patients had self-reported and EMR data for the preoperative time point, 38 of the 45 (84%) patients at 4 months, and 56% patients at 12 months for a total of 108 pairs of data. At the time of this study, 20 of the 45 enrolled patients had not yet reached their 12-month postoperative point and thus had not yet completed their 12-month postoperative medication log. Patient characteristics of the 45 preoperative patients are included in Table 2. Indication for amputation in this patient population included osteomyelitis (33%), trauma (22%), malignancy (16%), chronic pain (14%), and other (15%). Self-reported data demonstrated an average of 2.6 (SD = 2.00) total pain medications at any one time compared with 3.27 (SD = 2.11) total pain medications in EMR data.

**Table 2.** Agreement between Patient Self-reported and EMR Medication Dosage Category for Anticonvulsants and Acetaminophen Medication Types

Anticonvulsant							Acetaminophen						
**Preoperative**							**Preoperative**						
	**Patient self-reported**							**Patient self-reported**					
**EMR**	**None**	**Low**	**Medium**	**High**	**%A**	**Κ**	**EMR**	**None**	**Low**	**Medium**	**High**	**%A**	**Κ**
**None**	27	1	0	0	96%	0.93	**None**	19	6	1	0	62%	0.41
**Low**	0	4	0	0			**Low**	2	8	0	0		
**Medium**	1	0	8	0			**Medium**	4	2	0	1		
**High**	0	0	0	4			**High**	0	0	1	1		
**4 months postoperative**							**4 months Postoperative**						
	**Patient self-reported**							**Patient self-reported**					
**EMR**	**None**	**Low**	**Medium**	**High**	**%A**	**Κ**	**EMR**	**None**	**Low**	**Medium**	**High**	**%A**	**Κ**
**None**	10	0	1	0	71%	0.67	**None**	12	3	0	0	45%	0.21
**Low**	1	3	1	1			**Low**	1	3	0	0		
**Medium**	2	3	10	0			**Medium**	9	6	2	1		
**High**	0	0	2	4			**High**	1	0	0	0		
**12 months postoperative**							**12 months Postoperative**						
	**Patient self-reported**							**Patient self-reported**					
**EMR**	**None**	**Low**	**Medium**	**High**	**%A**	**Κ**	**EMR**	**None**	**Low**	**Medium**	**High**	**%A**	**Κ**
**None**	6	0	1	0	64%	0.58	**None**	11	1	0	0	52%	0.17
**Low**	0	4	0	1			**Low**	2	2	0	0		
**Medium**	2	0	4	3			**Medium**	6	2	0	0		
**High**	0	0	2	2			**High**	0	1	0	0		

Overall, medication use was less frequently indicated in self-reported records than the EMR across all three pain medication types: opioids, anticonvulsants, and OTCs. Only 37% (n = 40) of self-reported data entries indicated opioid use, whereas 49% (n = 53) of EMR data entries demonstrated opioid use. Fifty-five percent (n = 59) of the self-reported data entries indicated anticonvulsant use compared with 57% (n = 62) of all EMR data entries designated anticonvulsant use. Lastly, only 38% (n = 41) of self-reported data entries designated acetaminophen use, and interestingly, EMR data reported a higher incidence of acetaminophen use with 51% (n = 56) of entries.

### Medication Usage Agreement between Self-reported and EMR Data

Percentage agreement and kappa coefficients for medication usage reported for each medication type are presented in Table 3. In summary, opioid use agreement between patient self-report and EMR data is high preoperatively (84%) and 12 months postoperatively (92%), but dipped at 4 months (68%). Anticonvulsant use agreement was high at all three time points (88%–96%), whereas acetaminophen use agreement was only fair to moderate for all time points (63%–73%). When all data were combined, there was substantial agreement for opioid use (81% agreement, kappa = 0.61), near-perfect agreement for anticonvulsants (92% agreement, kappa = 0.83), and only fair agreement for acetaminophen (68% agreement, kappa = 0.36).

**Table 3.** Agreement between Patient Self-reported and EMR Medication Dosage Category for Opioids

	Low Dose	Medium Dose	High Dose
Acetaminophen	≤1925 mg	1926–3249 mg	≥3250 mg
Opioid (MME)	≤36 mg	36–120 mg	≥120 mg
Anticonvulsant: Carbamazepine	≤400 mg	401–799 mg	≥800 mg
Anticonvulsant: Gabapentin	≤900 mg	899–2699 mg	≥2700 mg
Anticonvulsant: Oxcarbazepine	≤600 mg	599–1499 mg	≥ 1500 mg
Anticonvulsant: Pregabalin	≤150 mg	149– 449 mg	≥450 mg

### Dose Category Agreement between Self-reported and EMR Data

Medication dose category agreement between self-reported and EMR data is presented in Tables 4 and 5, showing trends similar to those of the medication usage agreements for each medication type.

**Table 4.** Dose Category for Each Medication

Opioids						
**Preoperative**						
	**Patient self-reported**					
**EMR**	**None**	**Low**	**Medium**	**High**	**%A**	**Κ**
**None**	22	0	2	1	78%	0.68
**Low**	3	4	1	0		
**Medium**	1	0	5	1		
**High**	0	1	0	4		
**4 months postoperative**						
	**Patient self-reported**					
**EMR**	**None**	**Low**	**Medium**	**High**	**%A**	**Κ**
**None**	16	0	1	0	58%	0.39
**Low**	5	1	2	0		
**Medium**	5	1	4	1		
**High**	1	0	0	1		
**12 months postoperative**						
	**Patient self-reported**					
**EMR**	**None**	**Low**	**Medium**	**High**	**%A**	**Κ**
**None**	13	0	0	0	84%	0.76
**Low**	1	4	2	0		
**Medium**	0	0	2	0		
**High**	0	1	0	0		

**Table 5.** Demographic Characteristics of the Patients Whose Medication Logs Were Included in This Study

	Preoperative Participants (n = 45)	12-month Postoperative Participants (n = 25)	*P*
Average age	57	54	>0.05
Sex			
Male	29 (64%)	14 (56%)	>0.05
Female	16 (36%)	11 (42%)	>0.05
Race/ethnicity			
White	41 (91%)	22 (88%)	>0.05
Black or African American	3 (7%)	2 (8%)	>0.05
Current marital status			
Married or living with significant other	31 (69%)	17 (68%)	>0.05
Widowed	3 (7%)	1 (4%)	>0.05
Divorced	3 (7%)	2 (8%)	>0.05
Single, never married	7 (16%)	4 (16%)	>0.05
Annual gross household income			
Less than $25,000	16 (36%)	8 (32%)	>0.05
$25,000–$49,999	14 (31%)	6 (24%)	>0.05
$50,000–$74,999	9 (20%)	5 (20%)	>0.05
$75,000–$99,000	2 (4%)	3 (12%)	>0.05
$100,000 or more	3 (7%)	1 (4%)	>0.05
Smokers			
Never smoker	16 (36%)	7 (28%)	>0.05
Current smoker	8 (18%)	4 (16%)	>0.05
Previous smoker	20 (44%)	13 (52%)	>0.05
History of drug abuse?	2 (4%)	2 (8%)	>0.05
History of depression?	16 (36%)	8 (32%)	>0.05
History of chronic pain?	29 (64%)	19 (76%)	>0.05
History of preexisting pain conditions?			
None	15 (33%)	6 (24%)	>0.05
Complex regional pain syndrome	3 (7%)	1 (4%)	>0.05
Fibromyalgia	2 (4%)	1 (4%)	>0.05
Diabetic/peripheral neuropathy	7 (16%)	7 (28%)	>0.05
Claudication	5 (11%)	4 (16%)	>0.05
Chronic wound pain	5 (11%)	4 (16%)	>0.05
Other	13 (29%)	6 (24%)	>0.05

When all time points were combined, there was moderate agreement for opioid dose category (72% agreement, kappa = 0.60), fair agreement in acetaminophen dose reporting (53% agreement, kappa = 0.27), and substantial agreement with anticonvulsant dose reporting (80% agreement, kappa = 0.76).

### Dose Correlation between Self-reported and EMR Data

Self-reported and EMR medication dosages are graphed for each patient for both opioid and anticonvulsant medication categories. [**See graph, Supplemental Digital Content 1,** which displays EMR daily morphine equivalent doses plotted against self-reported doses for (A) all medication, (B) preoperative, (C) 4 months postoperative, and (D) 12 months postoperative. http://links.lww.com/PRSGO/C893.] **[See graph, Supplemental Digital Content 2,** which displays EMR anticonvulsant doses plotted against self-reported doses for (A) all medication, (B) preoperative, (C) 4 months postoperative, and (D) 12 months postoperative. http://links.lww.com/PRSGO/C894.] Spearman correlation coefficient was calculated for each medication type. Correlations for opioid self-reported and EMR doses were 0.72 for preoperative, 0.49 for 4 months postoperative, 0.80 for 12 months postoperative, and 0.65 for total records. Correlations for anticonvulsant self-reported and EMR doses were overall higher: 0.92 for preoperative, 0.73 for 4 months postoperative, and 0.61 for 12 months postoperative. Correlations for acetaminophen self-reported and EMR doses were overall lower: 0.38 for preoperative, 0.22 for 4 months postoperative, and 0.05 for 12 months postoperative. Combined across all time points, anticonvulsants and opioids had a correlation of 0.65, and acetaminophen had a correlation of 0.32.

## DISCUSSION

This study uniquely focuses on patients who have sustained limb loss, which is less often discussed in review of pain medication usage. Patients with amputations have a complicated relationship with pain, experiencing acute pain at times of surgical procedures as well as possessing a higher risk of developing chronic pain conditions such as neuroma and phantom limb pain.^20–22^ These patients are also at further risk of mental health conditions such as depression, anxiety, and posttraumatic stress disorder^23,24^ with these comorbidities being linked to chronic pain development and prescription opioid abuse.^21,25^ As a result, pain medication use in patients with amputations differs from the typical surgical populations and thus, must be studied. Expanding on the limited available literature, this study examines medication agreement between two sources of pain medication use data, building a foundation for the design and interpretation of future studies in this topic. As more emphasis is focused on the amputation population in recent literature, this foundation becomes increasingly important.

A 2021 systematic review by Brune and colleagues identified 120 studies that examined medication intake agreement between self-reported and other data sources and found that agreement ranged from poor to excellent.^26^ The populations of the various studies were diverse with many studies focused on cancer populations (17%),^26^ but no studies examined surgical populations.^26^ We attribute the wide range of agreement to the diversity of patient populations included in the Brune review. In general, analgesic medications have lower agreements between patient self-reported and other forms of data (eg, EMR, pharmacy records, prescription monitoring systems) compared with other medication types.^27–30^ Comparatively, the analgesic agreement between patient self-reported and EMR data presented within this study showed mostly substantial agreement except in the case of OTC acetaminophen. Previous studies grouped OTC medications within the analgesic categories, contributing to a decreased overall agreement,^27–30^ whereas this study examined agreement in separate categories of medications. Poor agreement of OTC medications between self-reported and EMR has been well documented in the literature,^31–34^ with one study by Stewart and colleagues listing OTC usage as the number one reason for discrepancies seen between self-reported and EMR medication data.^32^ Interestingly, the studies often describe EMR denoting less OTC medication,^31–33^ whereas the results of the current study indicate higher EMR acetaminophen logging compared with patient self-reporting. This observed difference could be due to several reasons, including institutional procedures and/or failure of the patients to self-report the medication.^32^

Kappa values of opioid agreement between self-reported and EMR have been reported to be rather low (0.35–0.46) in previous studies.^35,36^ It should be noted, however, that there is a paucity of literature examining opioid use agreement between self-reported and other data sources, especially in the surgical or limb amputation population. In general, our study found substantial agreement across combined time points for self-reported and EMR opioid data, suggesting that both datasets are adequate sources of information for accurate assessment of medication use. Furthermore, high agreement was seen in the population of patients with amputations, which may suggest less bias or better care coordination compared with other patient populations.

Our study showed substantial agreement at both preoperative and 12-month postoperative times for opioid usage at 84% and 92%, and dose category at 72% and 84%, respectively. However, at the 4-month postoperative visit, agreement was lower at 68% for opioid usage and 58% for dose category. Similarly, correlation of opioid dose across self-reported and EMR data also decreased at the 4-month postoperative visit. The discrepancies at 4 months were primarily due to higher EMR opioid doses compared with self-reported data. A few explanations could be applied to these findings. In the literature, most common reasons for this type of discrepancy are lack of reporting by the patient, patients stopping medication usage, or changes and/or discontinuation at recent clinic visits.^32^ It is important to note that the pain medication in the anticonvulsant category did not experience the same divergence of agreement at 4 months, making reasons like changes/discontinuation at recent clinic visits and lack of patient reporting less likely. A probable explanation for the higher divergence at the 4-month postoperative time point is that patients are likely discontinuing opioid use at home before the clinic visit given the resolution of acute surgical pain. Opioids are often prescribed as PRN for surgical pain management and are reflected as such in the EMR system. This type of prescribing did not have much impact on the high agreement between preoperative and 12-month postoperative medication intake, but likely impacted the 4-month medication reporting greatly due to self-discontinuation of the opioid medication. Thus, the time lag between self-discontinuation and the EMR records can explain higher use reported in the EMR. Although self-reported and EMR medication intake give similar information at preoperative and 12-month postoperative visits, data reflecting accurate medication intake for timepoints immediately after the expected end of acute surgical pain (3–4 months postoperative) may require either self-reported or a combination of self-reported and EMR data due to the large impact of discontinued pain medication on decreasing agreement.

This study reveals that EMR is not a great substitute for self-reported patient medication intake data in certain circumstances, particularly the 4-month postoperative period. Current EMR systems are limited in that they merely reflect prescribed medications without the ability to implement real-time and accurate analgesic medication use data. However, improving correlation between EMR and patient-reported data would be laborious and complicated, requiring a method for patients to log their analgesic medication intake daily and for that data to be easily transmittable into the EMR. One might suggest more pointed history-taking techniques at every clinical visit, with care to ask clarifying questions about dosing and frequency to calculate exact medication intake. Providers could then edit the EMR data to reflect patient-reported use and include this information in every clinical visit note for record keeping.

Overall, this study emphasizes the limitations of the EMR system when it comes to critically appraising patient analgesic medication use for clinical decision-making or study result interpretation. This is of particular importance in situations where self-reported patient medication intake data are not collected or unobtainable (ie, retrospective clinical studies). Future studies should explore the reasons for the divergence between self-reported and EMR medication intake data as well as methods for improving agreement systematically.

## LIMITATIONS

The largest limitation of this study relates to the fact that the patients within this study were primarily enrolled in a prospective study looking at the impact of an innovative surgical procedure for treatment and/or prevention of chronic pain. Thus, the data collection method and patient population were limited by this primary goal. First, data collection was constricted to three time points: preoperative, 4 months postoperative, and 12 months postoperative. Unfortunately, we were not able to assess medication intake more frequently in the ideal 0–4 months postoperative time frame, as most pain medications would have most likely been prescribed during this time. Second, our patient population of individuals who have sustained an amputation included a relatively small sample size of only 45 patients. We further acknowledge the PRN dosing information, and our interpretation of these data could explain some of the discrepancies in medication reporting agreements and is a limitation of this study.

It should finally be noted that our surgical population has a higher proportion of tobacco use and preexisting pain conditions (Table 2) that may confound pain experiences in these subjects. However, our results make no assertions about magnitude of pain medication use. This study does not examine pain outcomes. We similarly emphasize that our data do not and cannot assert superiority of one dataset over the other. Our study does not examine true accuracy of medication intake, but rather agreement in medication reporting between the EMR and survey data. In fact, accuracy of pain medication intake would be extremely challenging to achieve without continuous patient supervision.

## CONCLUSIONS

In conclusion, this study explores the agreement of different datasets of multimodal pain medication use in a surgical amputation population. Overall, self-reported data correspond greatly with EMR data, suggesting that either modality can be utilized without fear of inconsistencies, apart from OTC medications. However, given the data divergence after the expected end of acute surgical pain, the use of either self-reported or a combination of datasets might be warranted to capture a comprehensive understanding of a patient’s actual medication consumption. Future opioid study design and informed clinical decision-making should involve deliberate decisions on data type selection because it impacts the experimental and clinical outcomes.

## DISCLOSURES

The authors have no financial interests to declare in relation to the content of this article. This study was supported by Congressionally Directed Medical Research Program Peer Reviewed Orthopaedic Research Program, grant W81XWH-17-1-0641, and National Institute of Arthritis and Musculoskeletal and Skin Diseases of the National Institutes of Health under Award Number P30 AR069620.

## ACKNOWLEDGMENT

We acknowledge Nathan Bryant, the pain clinical pharmacist specialist, for his help in the development of medication dose categories.

## Supplementary Material
