# Supplementary material for: Agreement between Patient-reported Pain Medication Use and Electronic Medical Record Data in Surgical Amputation Patients
Source: Plast Reconstr Surg Glob Open. 2023 Nov 27;11(11):e5415. doi: 10.1097/GOX.0000000000005415 (PMC10681441; doi:10.1097/GOX.0000000000005415)
Supplement: Supplementary file 1 [file gox-11-e5415-s001.pdf]

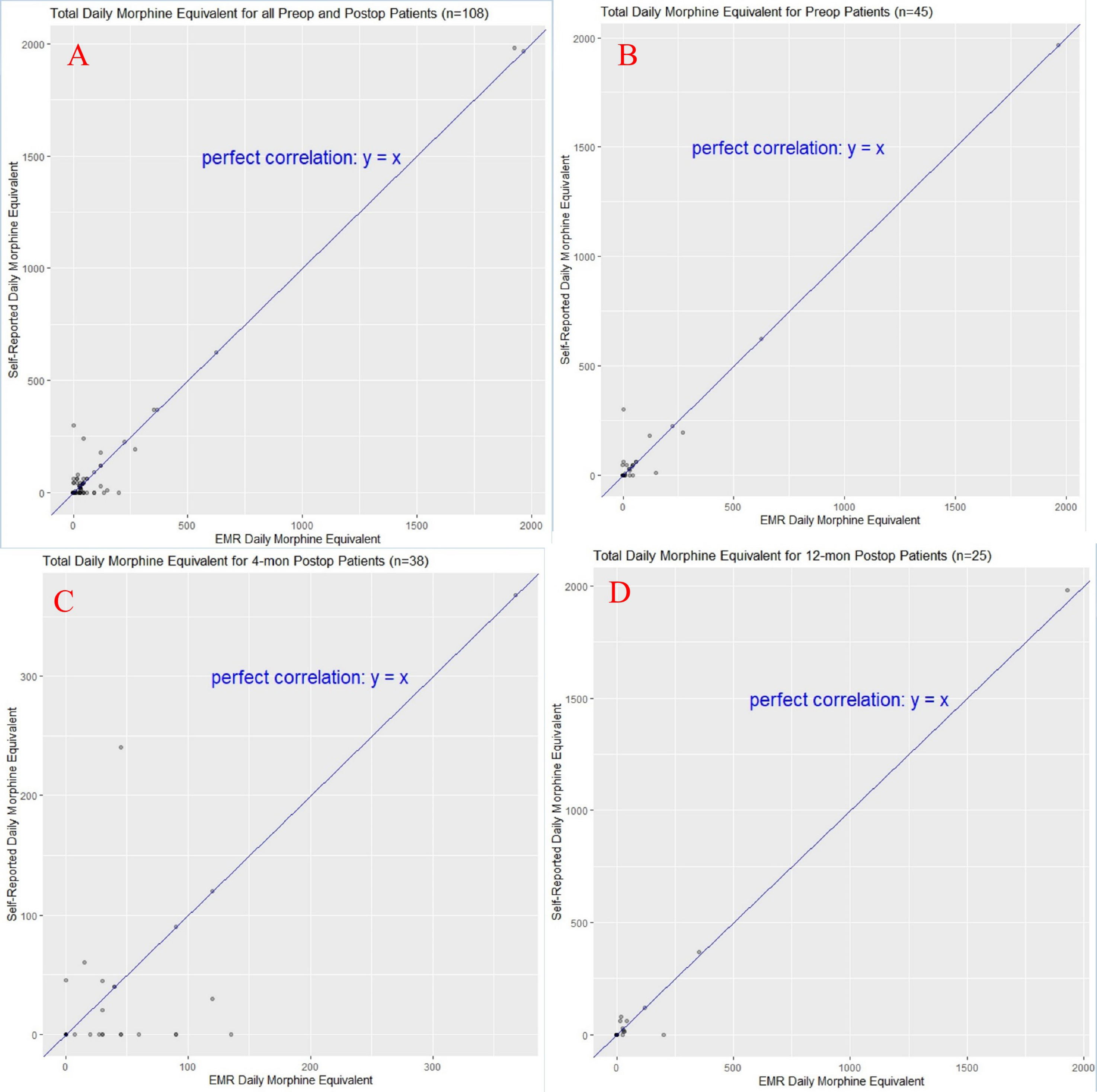
A
Total Daily Morphine Equivalent for all Preop and Postop Patients (n=108)
perfect correlation: y = x
Self-Reported Daily Morphine Equivalent
EMR Daily Morphine Equivalent
0
500
1000
1500
2000
B
Total Daily Morphine Equivalent for Preop Patients (n=45)
perfect correlation: y = x
Self-Reported Daily Morphine Equivalent
EMR Daily Morphine Equivalent
0
500
1000
1500
2000
C
Total Daily Morphine Equivalent for 4-mon Postop Patients (n=38)
perfect correlation: y = x
Self-Reported Daily Morphine Equivalent
EMR Daily Morphine Equivalent
0
100
200
300
D
Total Daily Morphine Equivalent for 12-mon Postop Patients (n=25)
perfect correlation: y = x
Self-Reported Daily Morphine Equivalent
EMR Daily Morphine Equivalent
0
500
1000
1500
2000
