# Supplementary material for: Agreement between Patient-reported Pain Medication Use and Electronic Medical Record Data in Surgical Amputation Patients
Source: Plast Reconstr Surg Glob Open. 2023 Nov 27;11(11):e5415. doi: 10.1097/GOX.0000000000005415 (PMC10681441; doi:10.1097/GOX.0000000000005415)
Supplement: Supplementary file 2 [file gox-11-e5415-s002.pdf]

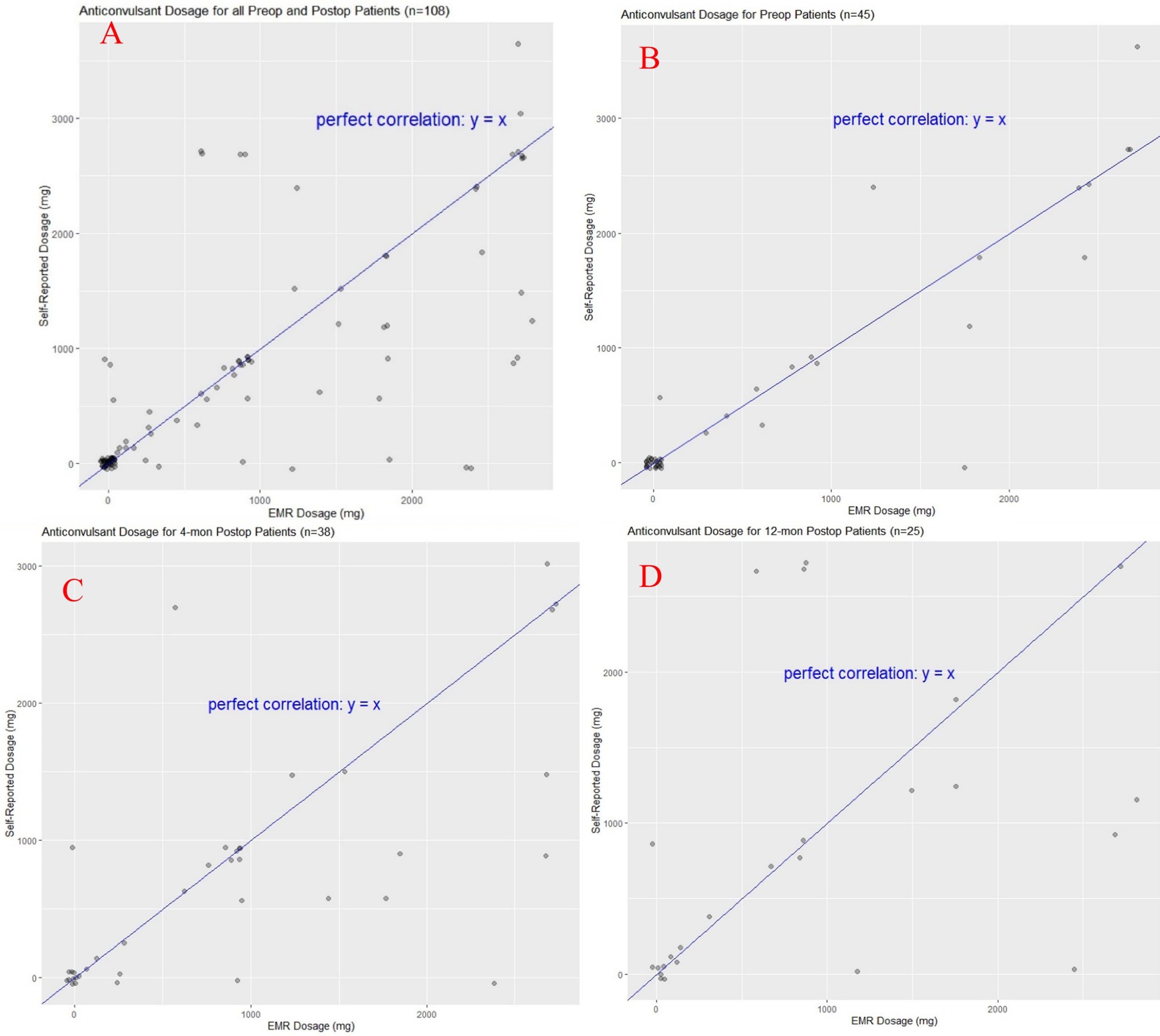

Anticonvulsant Dosage for all Preop and Postop Patients (n=108)
A
perfect correlation: y = x
Self-Reported Dosage (mg)
EMR Dosage (mg)
0
1000
2000
3000
Anticonvulsant Dosage for Preop Patients (n=45)
B
perfect correlation: y = x
Self-Reported Dosage (mg)
EMR Dosage (mg)
0
1000
2000
3000
Anticonvulsant Dosage for 4-mon Postop Patients (n=38)
C
perfect correlation: y = x
Self-Reported Dosage (mg)
EMR Dosage (mg)
0
1000
2000
3000
Anticonvulsant Dosage for 12-mon Postop Patients (n=25)
D
perfect correlation: y = x
Self-Reported Dosage (mg)
EMR Dosage (mg)
0
1000
2000
